# Chemical Assault Burns Leading to Multi-organ Complications and End-Organ Damage: A Case Series

**DOI:** 10.7759/cureus.105974

**Published:** 2026-03-27

**Authors:** Alizah Khan, Tasnim Mustafa, Shiza Ali, Yasmin H Kazim

**Affiliations:** 1 Clinical Sciences Department, Dubai Medical University, Dubai, ARE; 2 Emergency Department, Rashid Hospital - Dubai Health, Dubai, ARE

**Keywords:** assault injury, caustic substance, disseminated intravascular coagulation (dic), full-thickness burn, multiple-organ failure

## Abstract

This report presents two cases of extensive chemical burn injuries following acute exposure. The first involved a 24-year-old previously healthy female who sustained deep dermal and full-thickness burns over 44% of total body surface area, including the face, neck, chest, and upper limbs. Her injuries were complicated by circumferential limb burns, ocular involvement with corneal damage, and rhabdomyolysis leading to early acute kidney injury. Management required airway protection, aggressive fluid resuscitation, serial debridements, staged skin grafting, and reconstructive surgery. After seven months of hospitalization, she was discharged with stable grafts, preserved limb function, partial visual impairment, and significant scarring. The second case involved a 33-year-old male with 38% full-thickness burns affecting the face, neck, and upper body, accompanied by airway compromise, metabolic acidosis, coagulopathy, rhabdomyolysis, and progressive renal failure necessitating dialysis. Early intubation, escharotomy, and intensive supportive care were instituted. He required prolonged ventilation and multiple grafting and contracture release procedures. After six months, he achieved full clinical recovery. These cases highlight that chemical burns exceeding 30% total body surface area (TBSA) can precipitate overt disseminated intravascular coagulation (DIC), rhabdomyolysis-driven acute kidney injury (AKI), and multi-organ dysfunction even in the absence of initial haemodynamic instability, underscoring the need for early biochemical surveillance protocols and proactive multidisciplinary escalation from the time of presentation.

## Introduction

Chemical burns are caused by corrosive agents like acids and alkali, which lead to extensive tissue damage [1]. Such burns require specialized treatment distinct from thermal burns. A review of the literature yields that chemical burns represent a small proportion of all burns worldwide, but the severity and morbidity tend to be greater than those of other burn types. Females were slightly more likely to be victims (50.76%), while males were more frequently the perpetrators (89.01%) [2]. Of the different substances involved in chemical burns, acids were the most commonly used, accounting for 70.36% of cases [2].

Chemical burns result in tissue damage primarily through protein denaturation, with the severity of injury influenced by factors such as the concentration and quantity of the chemical, duration of exposure, and its specific mechanism of action, including reduction and oxidation reactions, corrosion, protoplasmic poisoning, vesication, and desiccation. Although the clinical presentation of chemical burns is broadly similar across different agents, the underlying mechanisms of tissue injury vary. Consequently, chemical burns have traditionally been classified into acids and alkalis. Acid burns typically cause tissue injury through protein denaturation, leading to coagulative necrosis, which tends to be localized and self-limiting. By contrast, alkaline burns result in liquefactive necrosis, allowing deeper tissue penetration and ongoing tissue damage with a more prolonged clinical course [3].

Burns involving more than 30% of the total body surface area (TBSA) are associated with significant hypovolemia and the release of inflammatory mediators, resulting in systemic effects, most notably a characteristic cardiovascular dysfunction known as burn shock. This represents a complex pathophysiological process involving both circulatory and microcirculatory impairment, leading to edema in burned as well as non-burned tissues. Despite prompt resuscitation and adequate fluid therapy, this state may not be completely reversible and is associated with substantial morbidity. Burn shock is characterized by inadequate tissue perfusion, resulting in impaired delivery of oxygen and nutrients and reduced clearance of metabolic waste products from affected tissues. Despite appropriate fluid resuscitation and restoration of preload, pulmonary and systemic vascular resistance increase, accompanied by myocardial depression. This further amplifies the inflammatory response and contributes to the development of multiple organ failure [4].

This report describes two cases of severe assault-related chemical burns that initially appeared to be superficial and limited, but ended up rapidly progressing, leading to early systemic complications including airway compromise, rhabdomyolysis, and multi-organ failure, despite prompt resuscitative measures.

## Case presentation

Case 1

A 24-year-old previously healthy woman was brought to the emergency department (ED) following a chemical assault with an unidentified corrosive substance involving the face, neck, chest, trunk, and upper limbs, approximately one hour prior to arrival. She reported a pain score of 10/10, most prominent in the right upper extremity. Past medical history was unremarkable.

**Figure 1:**
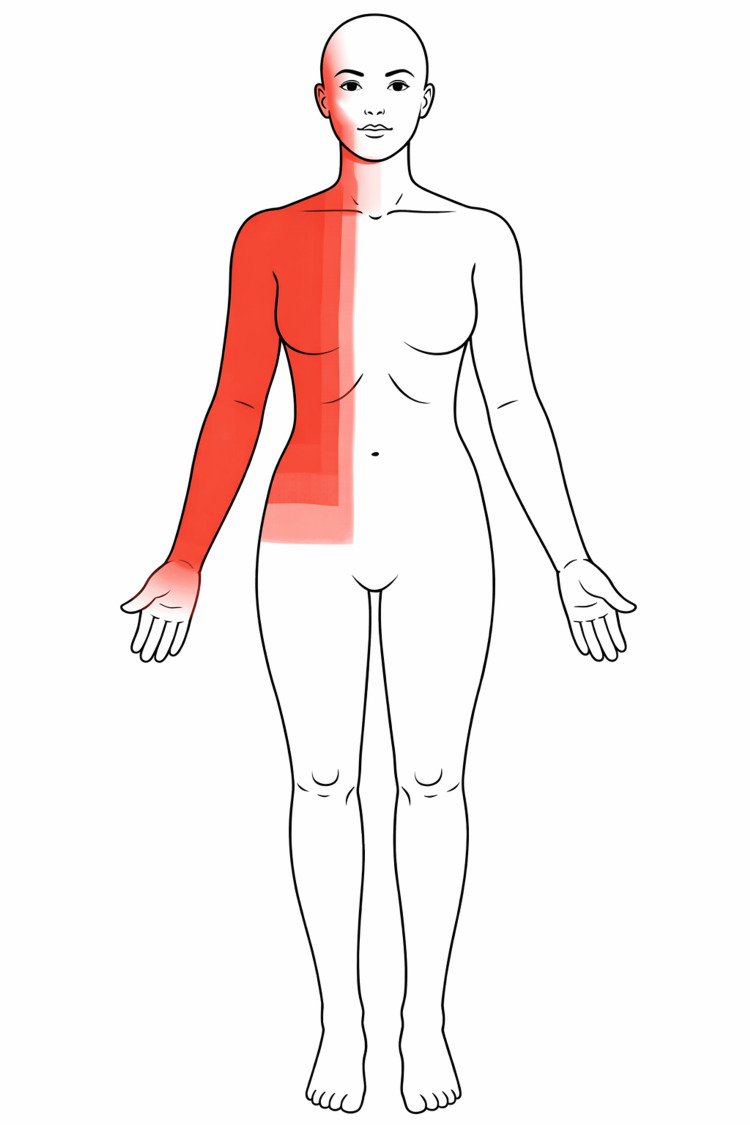
Anterior schematic representation demonstrating the extent of chemical burn involvement (40–44% total body surface area (TBSA)) affecting the right side of the face, eye, neck, anterior trunk, and upper limb.

On presentation, she was awake, oriented, and distressed but hemodynamically stable, with an oxygen saturation of 97% on room air. Clinical examination revealed mixed deep dermal and full-thickness chemical burns involving approximately 40-44% TBSA, predominantly affecting the right side of the face, neck, anterior chest wall, including the right breast and right upper limb. Head and neck examination demonstrated extensive facial and neck burns with involvement of the right eye, including corneal burns and marked upper eyelid edema. A circumferential burn of the right upper limb was noted with preserved pulses on initial assessment; however, given progressive and refractory pain suggestive of rising compartment pressure, bilateral escharotomy of the right upper limb was performed (Figure 1). As escharotomy failed to provide adequate pain relief and given the extensive distribution of face and neck burns, raising concern for progressive airway edema, the decision was made to proceed with early endotracheal intubation to electively secure the airway prior to the development of progressive airway edema and a compromised airway.

Intravenous fluid resuscitation was initiated with lactated Ringer’s solution according to the Parkland formula (4 mL × kg × %TBSA), with a calculated 24-hour requirement of approximately 8.3-9.2 litres. Urinary catheterisation shortly after admission drained cola-coloured urine, raising concern for rhabdomyolysis. Given the pigmenturia, urine output was targeted above 60 mL/hour (0.5-1 mL/kg/hour) to facilitate myoglobin clearance and protect renal tubular function, and fluid rates were titrated accordingly. This target was consistently achieved throughout the resuscitative phase, and despite early high-anion gap metabolic acidosis, renal function remained preserved during the initial admission period. Table 1 reports the initial laboratory findings.

**Table 1:** Case 1 laboratory findings

Parameter	Result	Reference range	Date
Hematology			
WBC	32.5 ×10³/µL	3.6–11.0 ×10³/µL	29/11/2024
Haemoglobin	14.7 → 8.3 g/dL (nadir)	12.0–15.0 g/dL	29/11/2024–Dec 2024
Platelet count	187 ×10³/µL	150–410 ×10³/µL	29/11/2024
Coagulation Profile			
Prothrombin time (PT)	25.6 s	11–14 s	29/11/2024
INR	2.30	0.8–1.1	29/11/2024
aPTT	83.7 s	28–41 s	29/11/2024
Fibrinogen	87 mg/dL	200–400 mg/dL	29/11/2024
D-dimer	>20.0 µg/mL FEU	<0.5 µg/mL FEU	29/11/2024
Blood Gas (Key Values)			
pH	7.327 → 7.388	7.35–7.45	29/11/2024–02/12/2024
HCO₃⁻	14.8 → 17.8 mmol/L	21–28 mmol/L	29/11/2024–02/12/2024
Lactate	3.29 mmol/L	0.5–1.6 mmol/L	29/11/2024
Renal Function			
eGFR	91.6 → 12.3 mL/min/1.73 m²	>90 mL/min/1.73 m²	29/11/2024–02/12/2024
Creatinine	0.90 → 4.78 mg/dL	0.7–1.3 mg/dL	29/11/2024–02/12/2024
CK	196 → 1,326 U/L	30–200 U/L	29/11/2024–01/12/2024

The patient was admitted to the intensive care unit (ICU) under a multidisciplinary team including intensive care, plastic surgery, and infectious disease specialists. Progressive anemia with hemoglobin declining to 8.3 g/dL, combined with worsening coagulopathy reflected by rising INR and falling fibrinogen, necessitated recurrent transfusions of packed red blood cells, fresh frozen plasma, and platelets, guided by serial hematological monitoring and operative bleeding requirements. Over the course of approximately six weeks, the patient received a total of 39 units of packed red blood cells, eight units of fresh frozen plasma, and 1 unit of platelets, administered across multiple sessions in response to progressive anemia and perioperative hemostatic requirements. As transfusions were distributed across this six-week period, it did not meet the criteria for activation of a massive transfusion protocol. Wound cultures identified *Pseudomonas aeruginosa* and AmpC-producing *Klebsiella pneumoniae*, and antimicrobial therapy (piperacillin-tazobactam) was de-escalated and tailored accordingly. Tracheostomy was later performed due to the anticipated prolonged treatment and recovery phases.

The clinical course unfolded across three broad phases. During the initial critical phase (November-December 2024), management focused on hemodynamic stabilization, correction of metabolic acidosis and coagulopathy, and serial surgical debridement of the trunk and all four limbs. Progression to definitive grafting was deferred until wound beds demonstrated clean, well-vascularised granulation tissue on serial assessment and coagulation parameters had reached a threshold deemed safe for operative intervention. The surgical reconstruction phase (January-March 2025) involved staged split-thickness skin grafting to the chest, abdomen, upper extremities, face, and lower limbs, followed by right axillary contracture release with Z-plasty and surgical release of a right upper eyelid contracture with graft placement, with satisfactory graft take. The final recovery phase (April-July 2025) involved further graft revisions, rehabilitation, and nutritional optimization in preparation for discharge.

**Figure 2:**
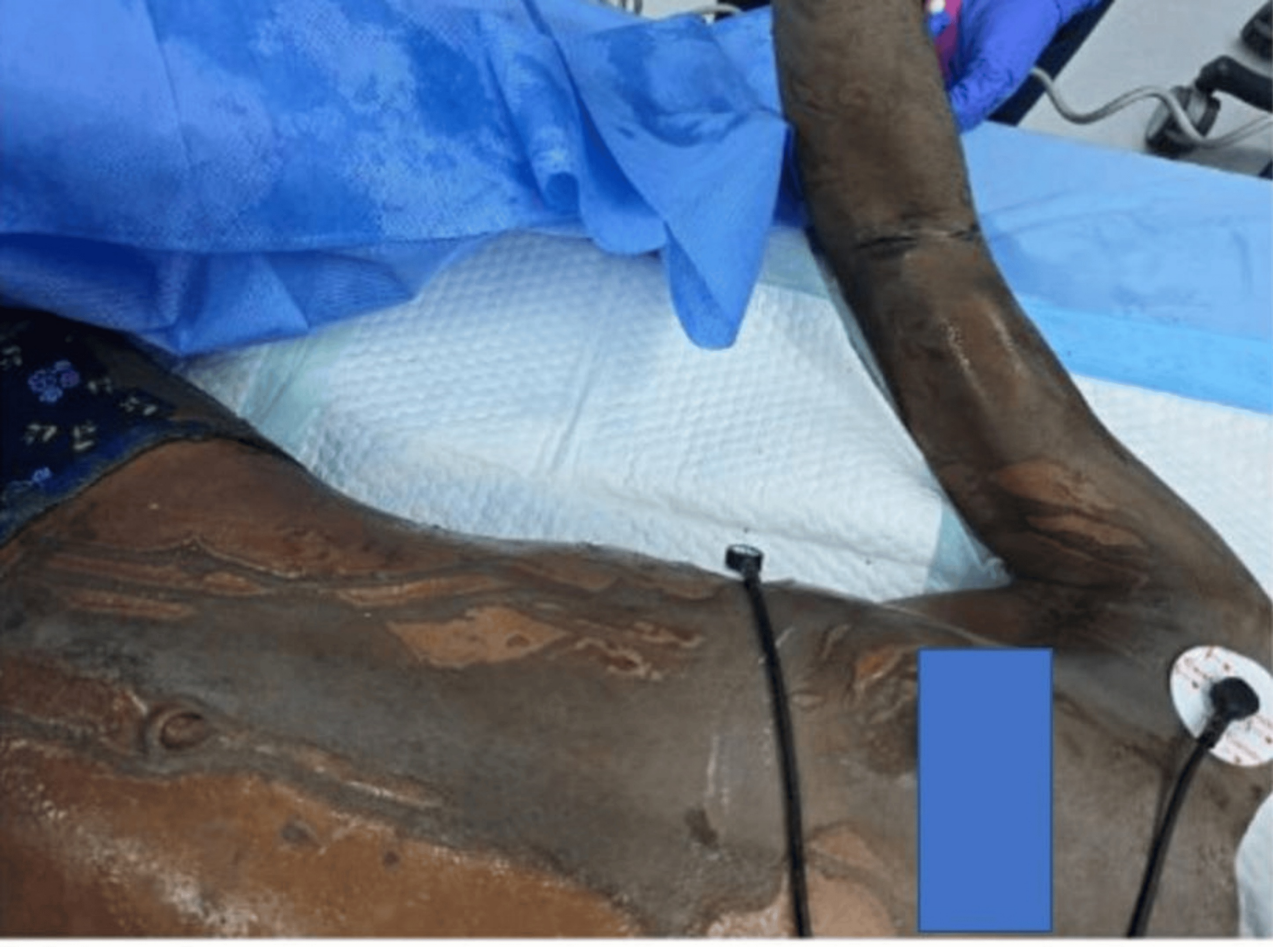
Clinical photograph of the right side of the chest, arm and abdomen demonstrating chemical burn injury with areas of deep dermal to full-thickness involvement, showing pale eschar formation consistent with deep tissue injury.

**Figure 3:**
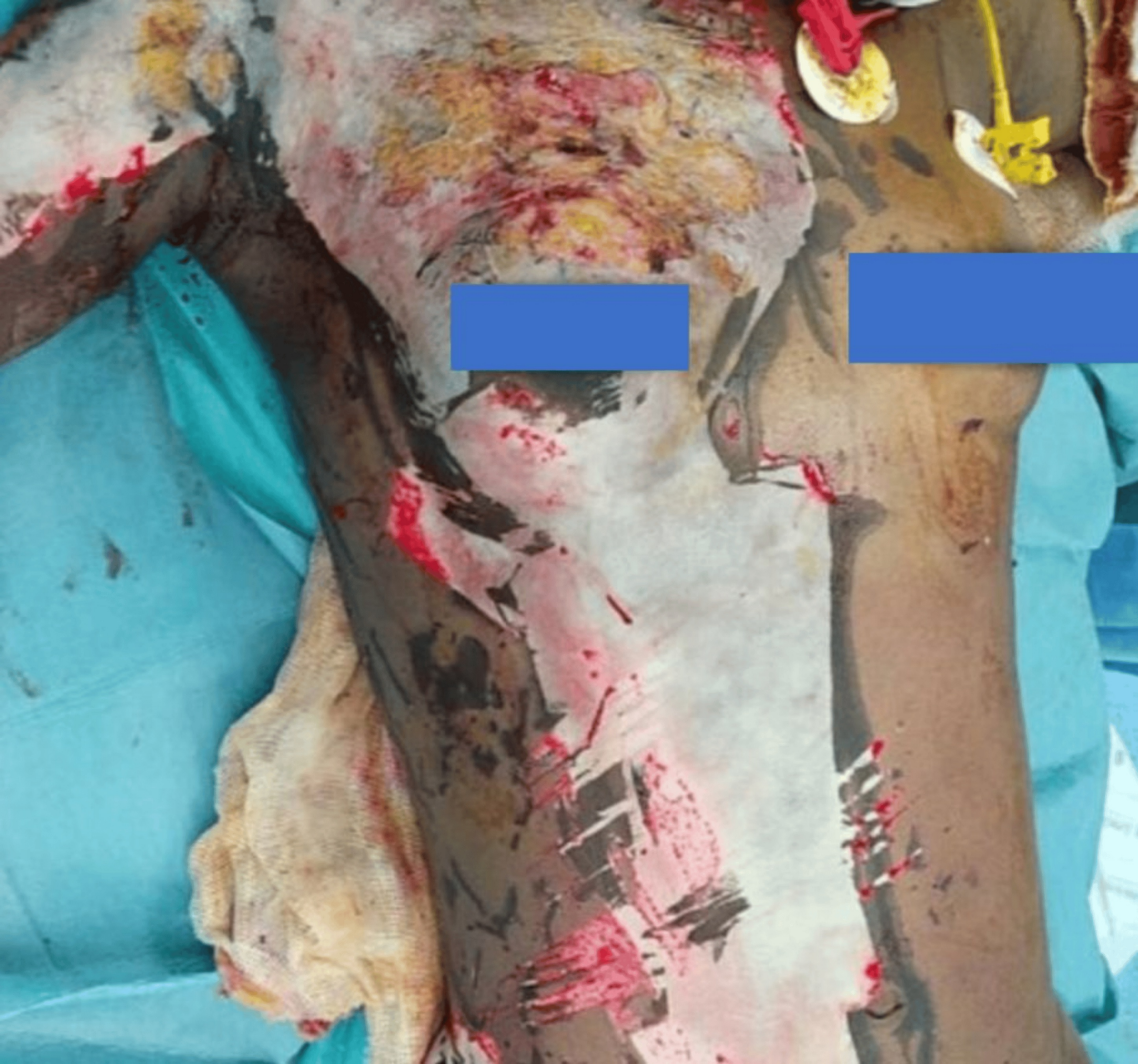
Early post-grafting appearance demonstrating split-thickness skin graft placement over the previously debrided burn area.

**Figure 4:**
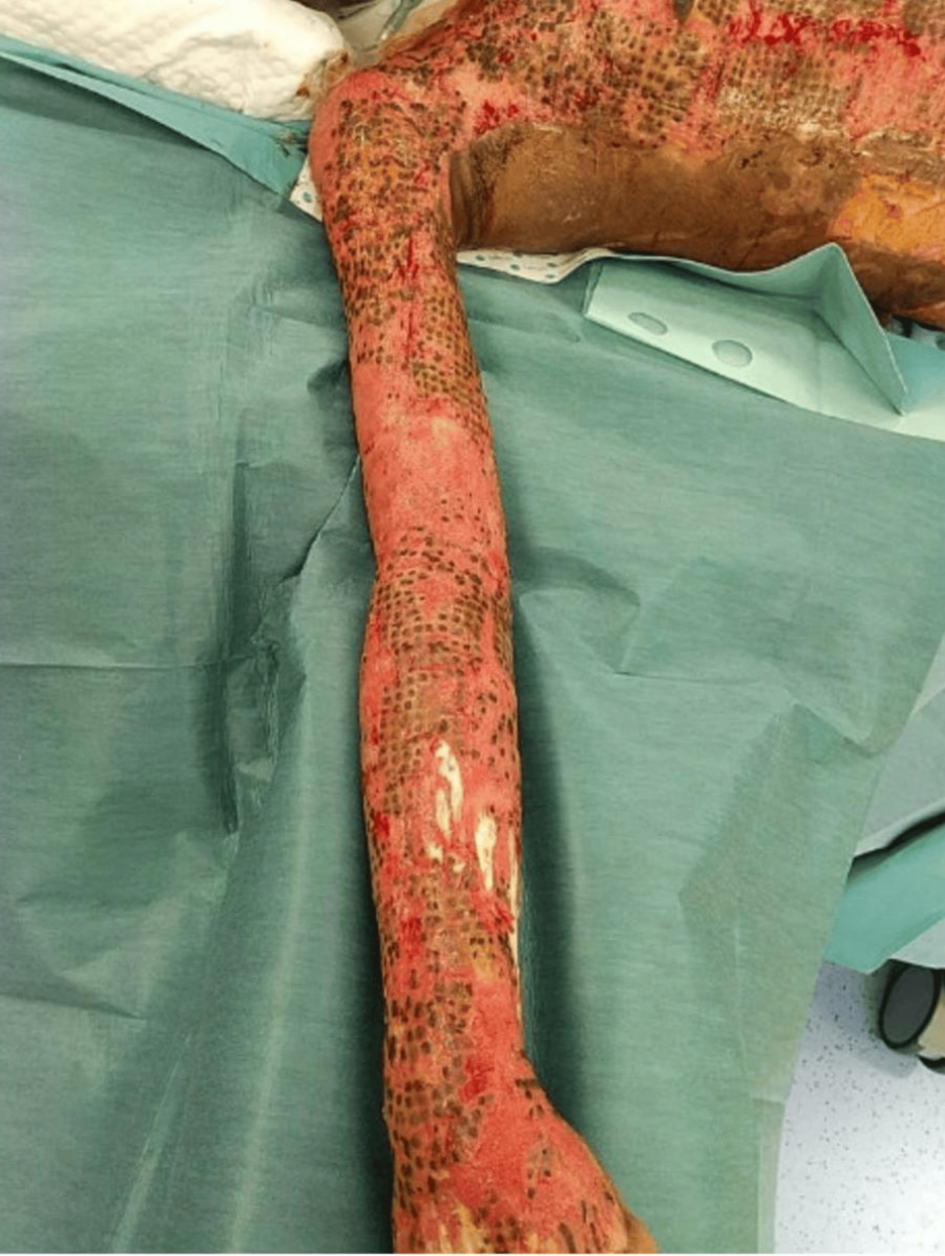
Post-reconstructive phase demonstrating satisfactory healing following split-thickness skin grafting, with stable graft uptake and progressive epithelialization.

After a seven-month hospitalization, the patient achieved stable wound healing with good graft integration. She was discharged in stable condition with extensive residual scarring, partial visual impairment of the right eye, and risk of recurrent contracture formation, but retained functional use of the affected limb, with planned outpatient plastic surgery follow-up (Figures 2-4).

Case 2

A 33-year-old previously healthy man was brought to the ED following an assault involving exposure to an unidentified caustic substance affecting the face, neck, and upper torso. The exposure occurred shortly before arrival. He similarly reported severe pain with a pain score of 10/10 and progressive neck tightness associated with increasing difficulty breathing. Past medical history was unremarkable.​

**Figure 5:**
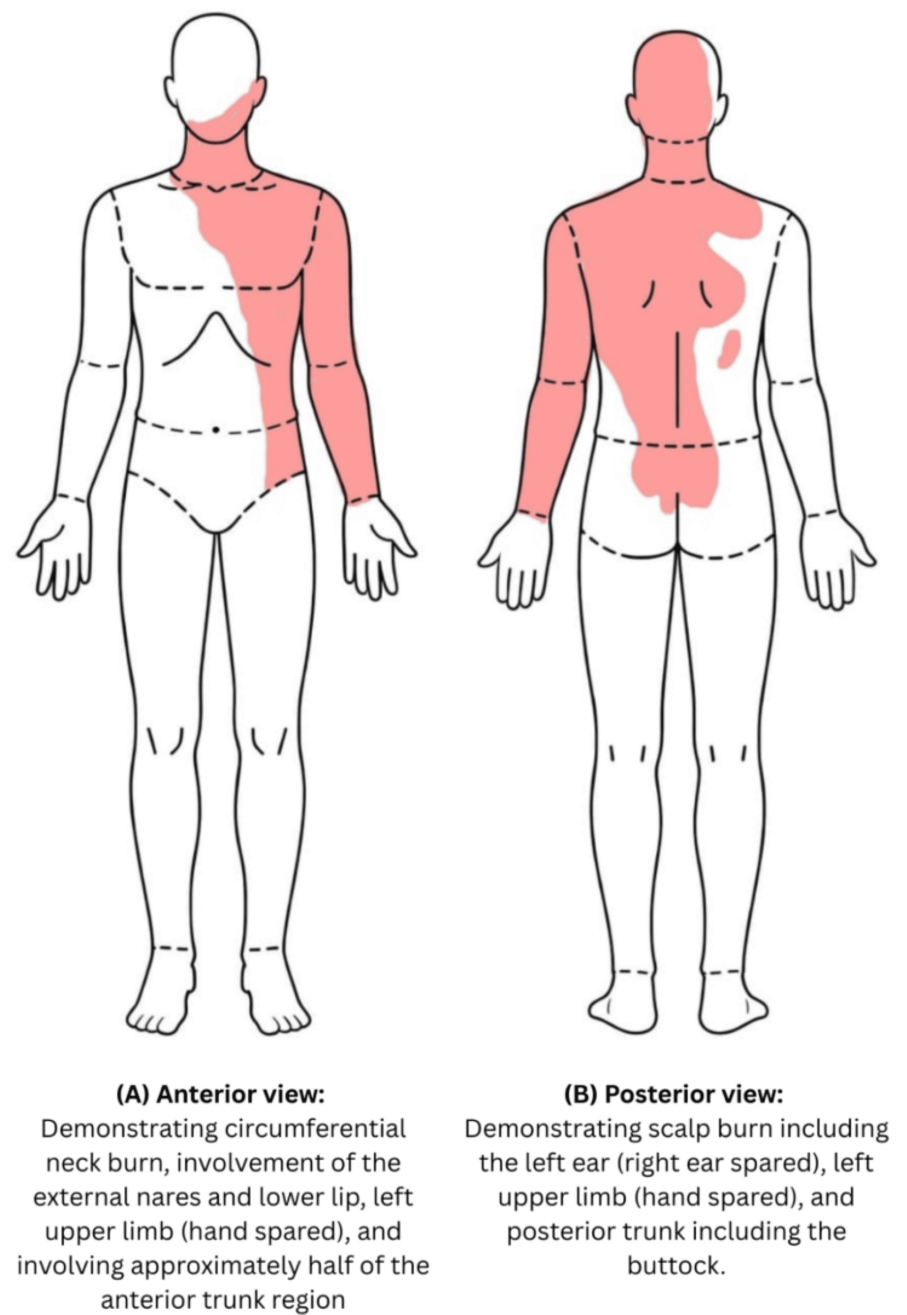
Burn distribution schematic: total body surface area (TBSA) ~38-40%

**Figure 6:**
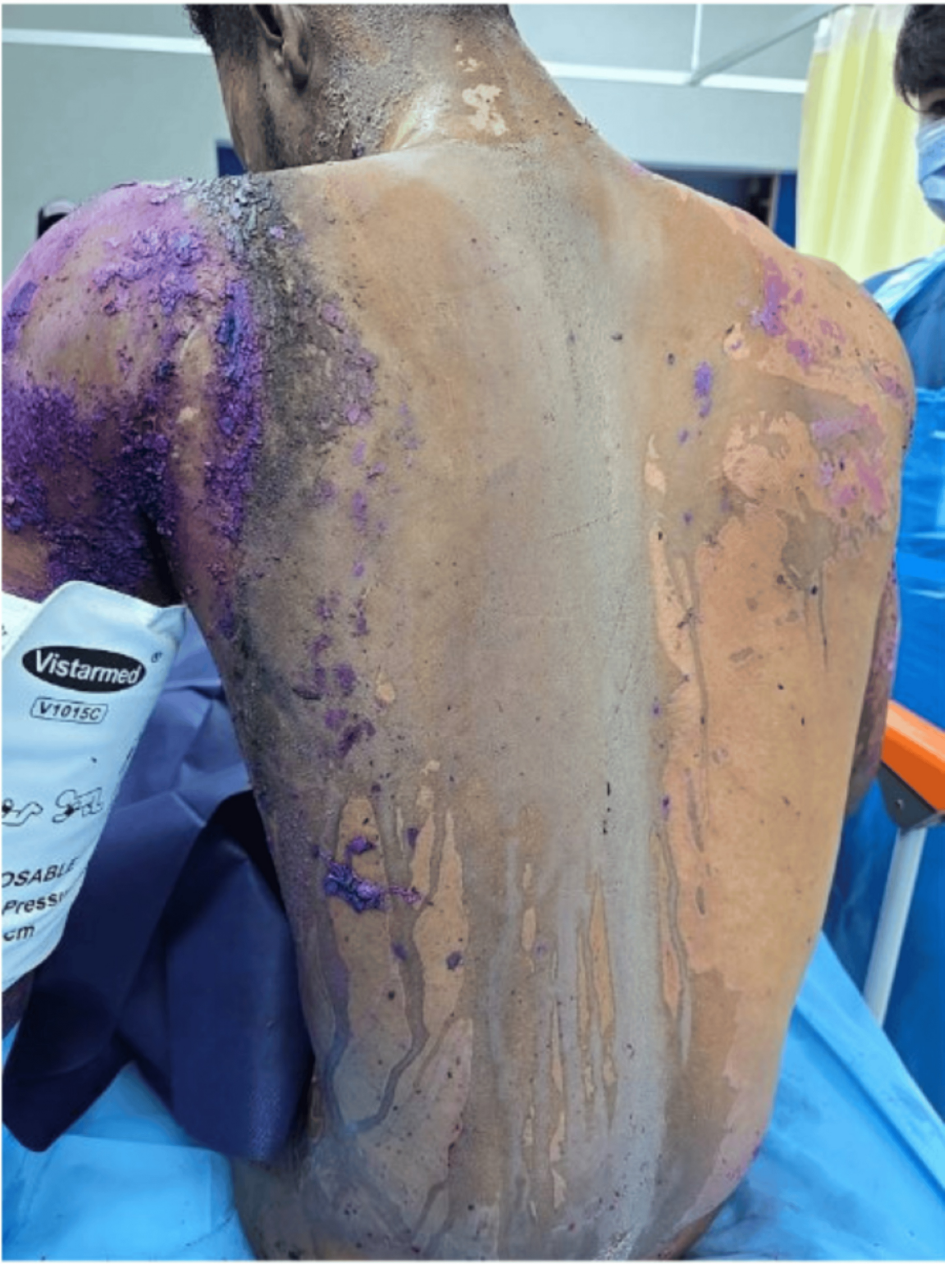
Posterior trunk chemical burn appearance on presentation

**Figure 7:**
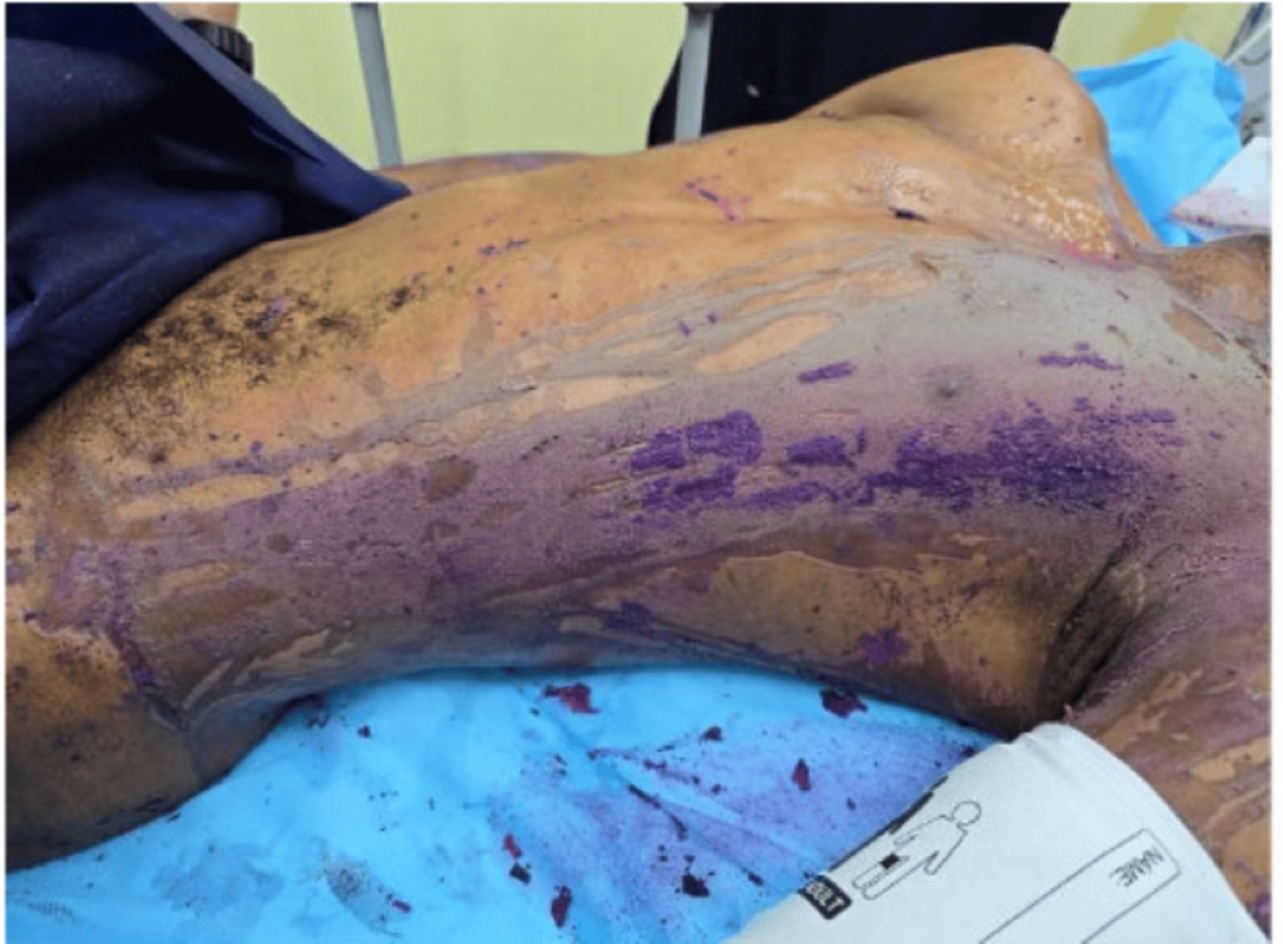
Anterior trunk chemical burn appearance on presentation

**Figure 8:**
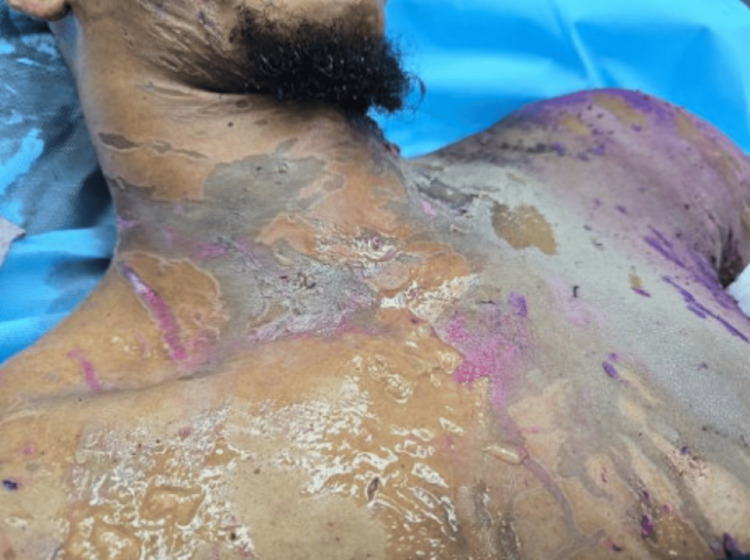
Circumferential neck burn on presentation

On initial assessment, he was alert, oriented, and visibly distressed but hemodynamically stable, and maintaining oxygen saturation at a 100% on room air. Physical examination revealed full-thickness chemical burns involving approximately 38% TBSA, predominantly affecting the face, neck, and upper body. Circumferential neck burns with tense eschar raised significant concern for impending airway obstruction (Figures 5-8).

**Figure 9:**
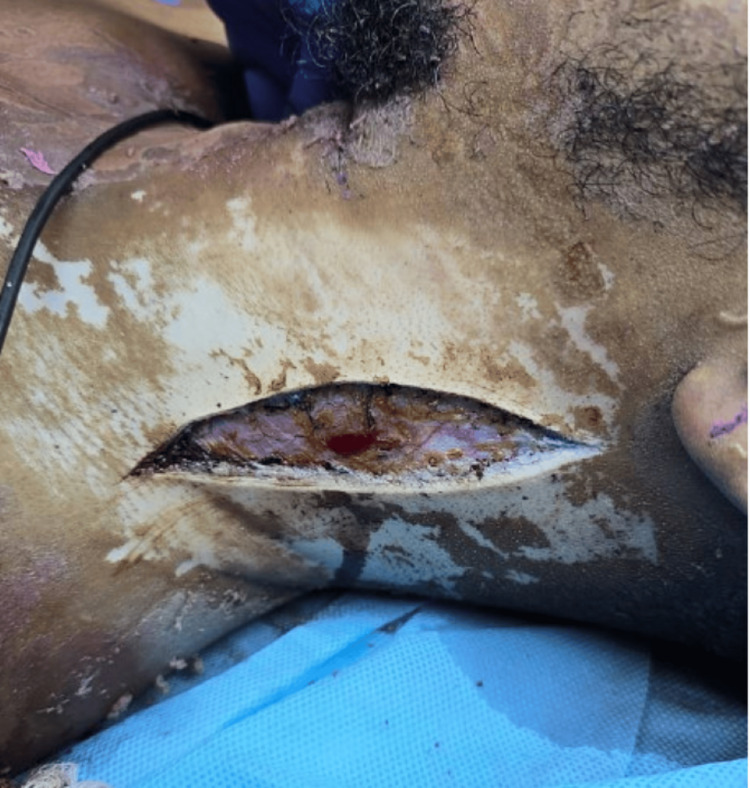
Left neck escharotomy performed for circumferential neck burn with concern for airway compromise.

Bilateral neck escharotomy was performed as a first-line intervention to decompress the circumferential cervical eschar and relieve extrinsic airway compression; however, despite escharotomy, the patient continued to experience progressive neck tightness and worsening respiratory difficulty (Figure 9).

As the clinical picture was consistent with deep edema extending beyond the eschar into the supraglottic and perilaryngeal soft tissues, a pattern not amenable to escharotomy alone, the decision was made to proceed with early endotracheal intubation before complete airway compromise developed. Urinary catheterization revealed cola-colored raising concern for rhabdomyolysis.

Intravenous fluid resuscitation was initiated with lactated Ringer’s solution according to Parkland-guided burn resuscitation protocols. Given the pigmenturia on admission, urine output was targeted above 70 mL/hour to facilitate myoglobin clearance and mitigate tubular precipitation. Despite adequate resuscitation achieving the target urine output, renal dysfunction progressed over the first week, with creatinine rising from 1.25 mg/dL to 6.60 mg/dL and CK peaking at 1,912 U/L by day 7. As creatinine continued to rise despite optimised fluid management, with concurrent severe metabolic acidosis and worsening fluid balance, the decision was made to initiate continuous renal replacement therapy (CRRT) rather than intermittent hemodialysis, given the patient’s hemodynamic vulnerability and the need for precise fluid control in the context of ongoing burn resuscitation and operative requirements. The failure to prevent AKI despite targeted resuscitation in this case, in contrast to Case 1, where renal function was preserved, likely reflects the greater systemic inflammatory burden, more severe rhabdomyolysis trajectory, and degree of metabolic acidosis at presentation in Case 2.

**Table 2:** Case 2 laboratory findings

Parameter	Result	Reference range	Date
Hematology			
WBC	12.7 → 11.7 ×10³/µL	3.6–11.0 ×10³/µL	29/11/2024–04/12/2024
Haemoglobin	14.7 → 6.2 g/dL (nadir)	12.0–15.0 g/dL	29/11/2024–04/12/2024
Platelet count	194 → 131 ×10³/µL	150–410 ×10³/µL	29/11/2024–04/12/2024
Coagulation Profile			
Prothrombin time (PT)	28.2 s	11–14 s	29/11/2024
INR	2.60	0.8–1.1	29/11/2024
aPTT	70.8 s	28–41 s	29/11/2024
Fibrinogen	132 mg/dL	200–400 mg/dL	29/11/2024
D-dimer	>20.0 µg/mL FEU	<0.5 µg/mL FEU	29/11/2024
Blood Gas (Key Values)			
pH	7.059	7.35–7.45	29/11/2024
HCO₃⁻	13.6 mmol/L	21–28 mmol/L	29/11/2024
Anion Gap	28 mmol/L	6–14 mmol/L	29/11/2024
Lactate	1.9 mmol/L	0.5–1.6 mmol/L	29/11/2024
Renal Function			
eGFR	78.0 → 10.6 mL/min/1.73 m²	>90 mL/min/1.73 m²	29/11/2024–02/12/2024
Creatinine	1.25 → 6.60 mg/dL	0.7–1.3 mg/dL	29/11/2024–02/12/2024
CK	292 → 1,912 U/L	30–200 U/L	29/11/2024–07/12/2024

The patient was managed in the ICU with mechanical ventilation, burn resuscitation, renal replacement therapy, correction of metabolic derangements, and multidisciplinary input from critical care, plastic surgery, nephrology, and infectious disease teams. Empiric broad-spectrum antimicrobial therapy was initiated with piperacillin-tazobactam (4.5g IV every 8 hours) given the high infectious risk in the context of extensive open burn wounds, and was subsequently de-escalated according to wound culture and susceptibility results. Coagulopathy was marked on admission, with an INR of 2.6, fibrinogen of 132 mg/dL, and D-dimer exceeding 20 µg/mL. Hemoglobin declined progressively to a nadir of 6.2 g/dL, and platelet counts fell from 194 to 131 × 10³/µL, reflecting ongoing consumptive coagulopathy. Transfusion of packed red blood cells was initiated when hemoglobin fell below 7 g/dL and when operative hemostasis was required; fresh frozen plasma and platelets were administered in response to worsening coagulation parameters and perioperative bleeding risk. Over approximately a three-month period, the patient received a total of 63 units of packed red blood cells, 24 units of fresh frozen plasma, and 1 unit of platelets. Transfusions were distributed across multiple episodes throughout this period and did not meet criteria for massive transfusion protocol activation, as no single episode involved transfusion of ten or more units of packed red blood cells within 24 hours. The cumulative transfusion burden reflects the sustained hemostatic instability and repeated operative interventions characteristic of extensive chemical burns with severe coagulopathy.

**Figure 10:**
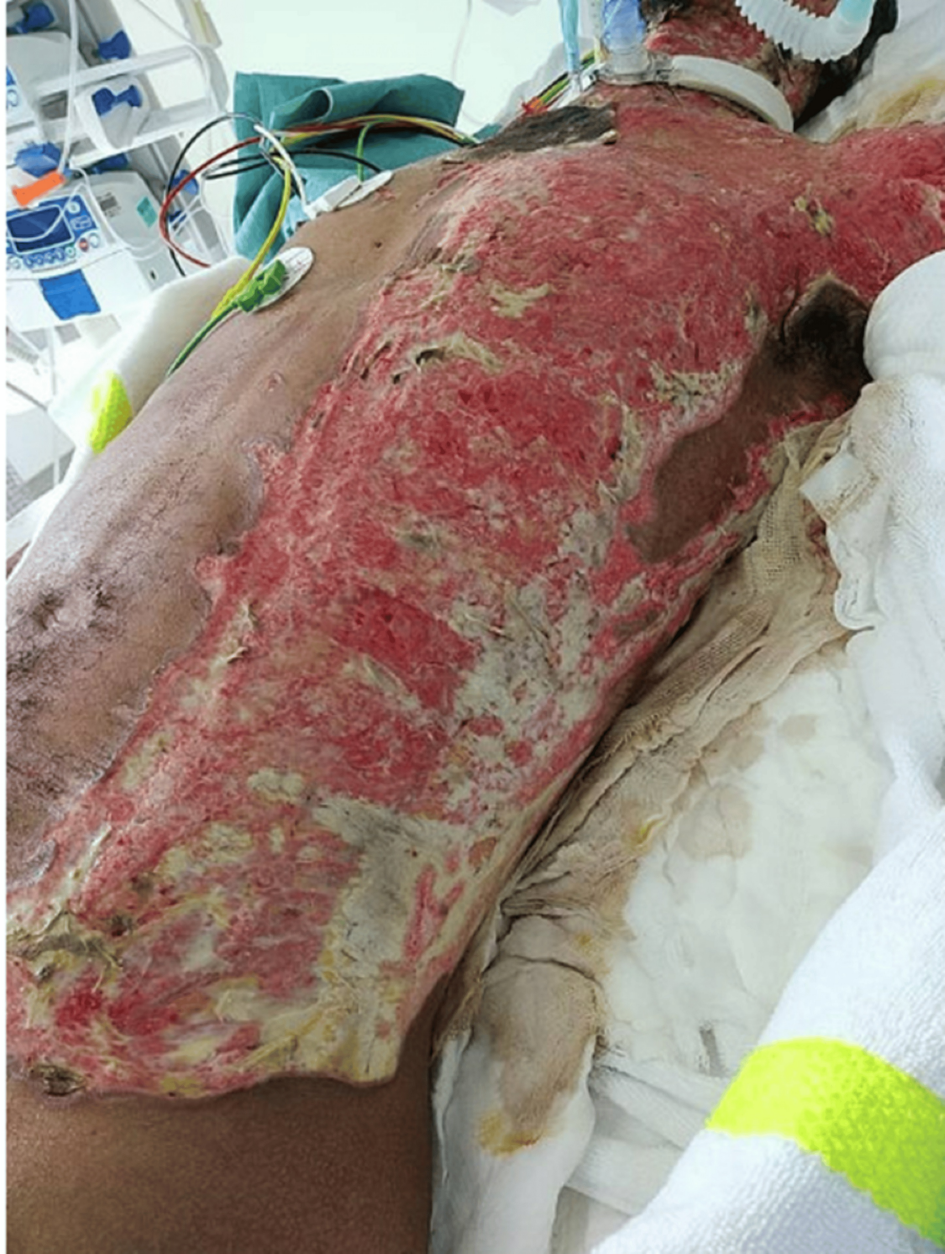
ICU photograph prior to grafting showing extensive mixed-depth burn involving the lateral chest wall/axilla and left lateral abdominal wall. The wound bed demonstrates erythematous granulation with adherent yellow slough/nonviable tissue, indicating the need for debridement and coverage.

**Figure 11:**
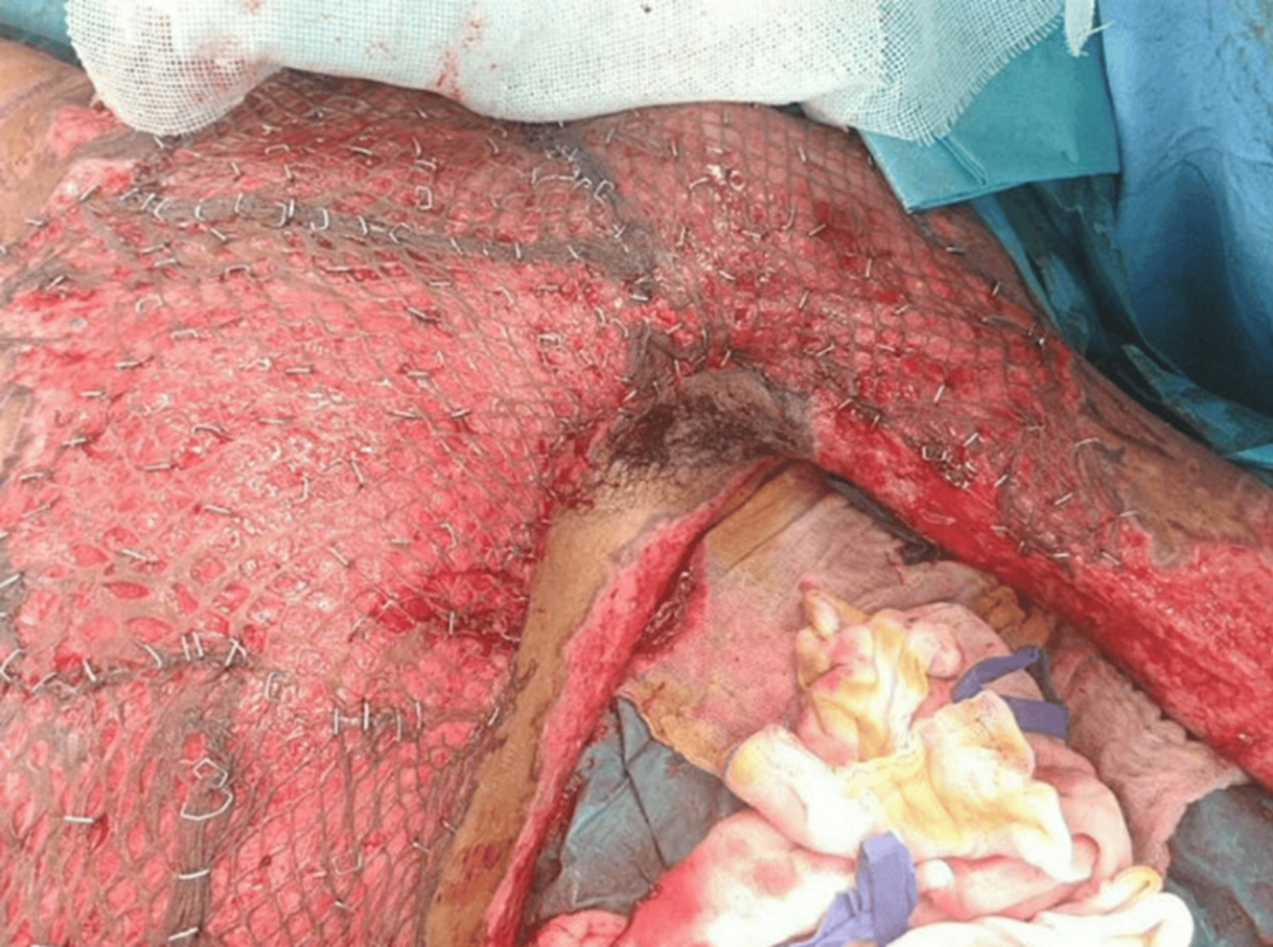
Intraoperative image showing freshly applied meshed split-thickness skin graft over the left lateral thoracoabdominal wall and axilla after burn excision. The graft is expanded and secured with staples, demonstrating uniform take and adequate defect coverage.

The clinical course similarly unfolded in three phases. The initial critical phase (November 2024-January 2025) was dominated by mechanical ventilation, CRRT, metabolic acidosis correction, and consumptive coagulopathy management, with serial surgical debridement performed once hemostatic parameters were sufficiently stabilised to allow safe operative intervention. Progression to definitive wound coverage was deferred until the wound bed demonstrated clean granulation tissue and systemic inflammatory and hematological parameters had reached operative thresholds. The reconstructive phase (February-March 2025) involved full-thickness skin grafting to the back and left arm and split-thickness autografting involving less than 30% TBSA. The late recovery phase (March-May 2025) included contracture release procedures, ongoing wound care, and rehabilitation (Figures 10, 11).

**Figure 12:**
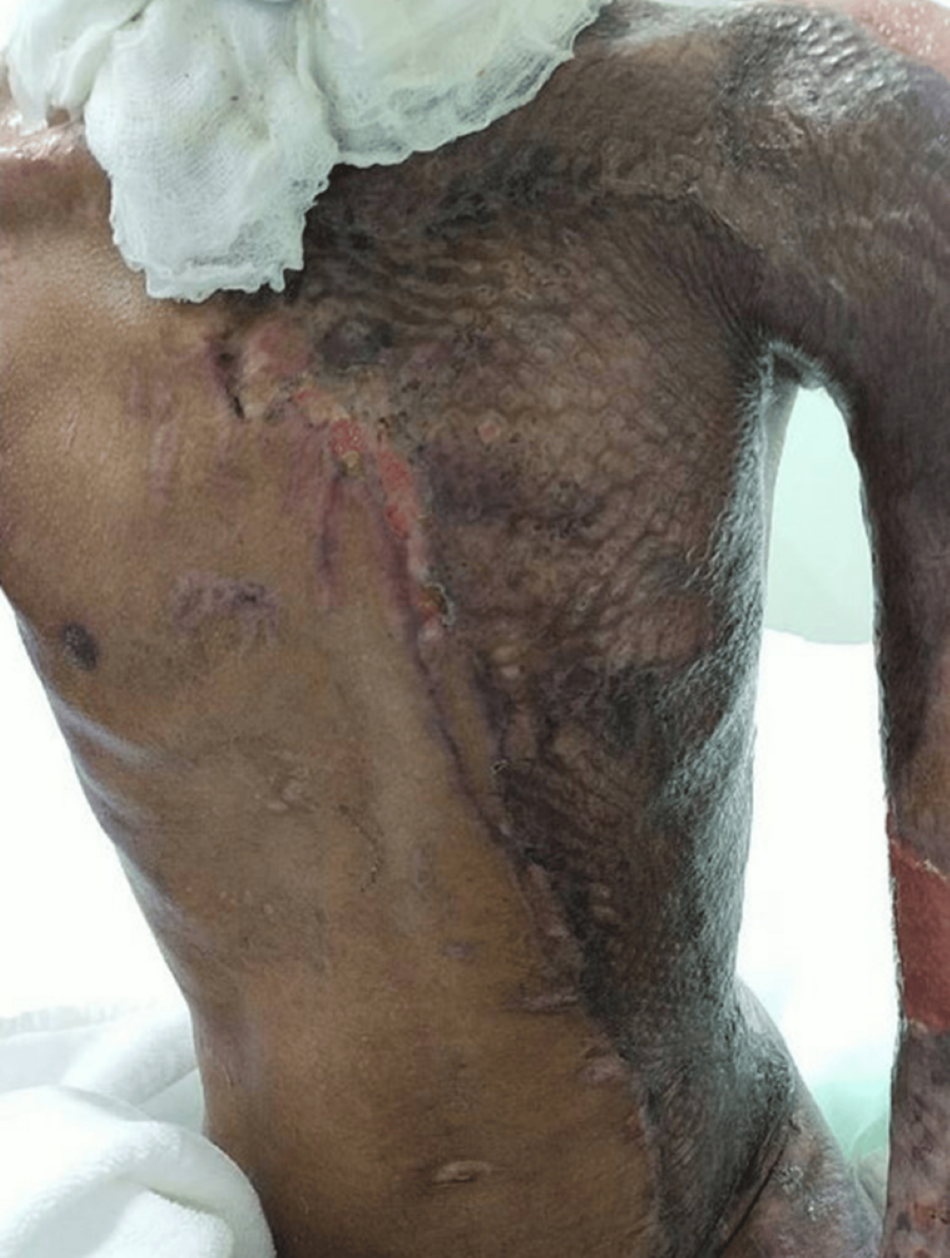
Late postoperative image showing a well-taken meshed split-thickness skin graft over the left thoracoabdominal wall and axilla. The graft demonstrates stable coverage with residual hyperpigmentation and mesh pattern visibility consistent with maturation.

**Figure 13:**
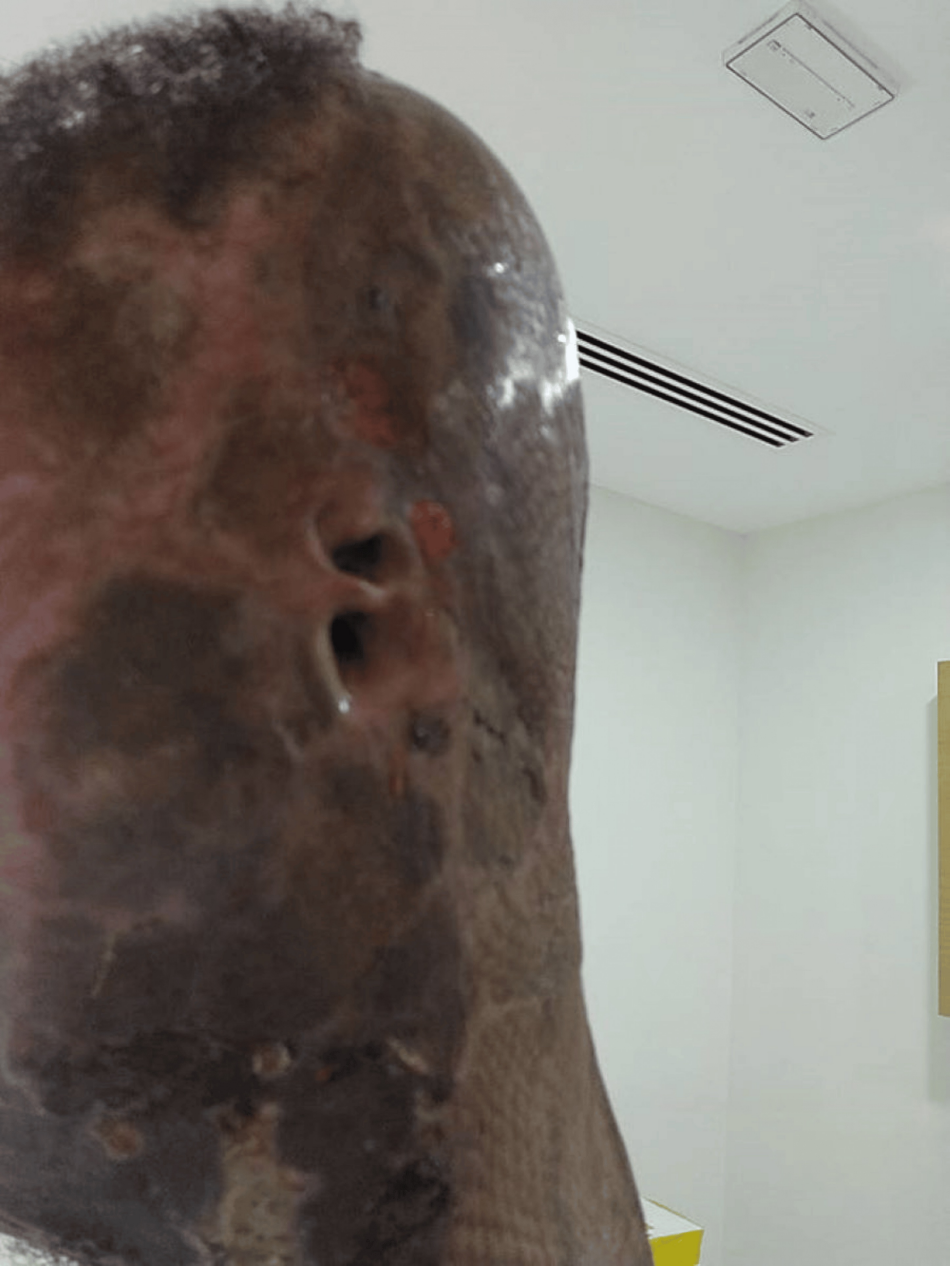
Image of the left side of the head and face evident of disfigurement.

**Figure 14:**
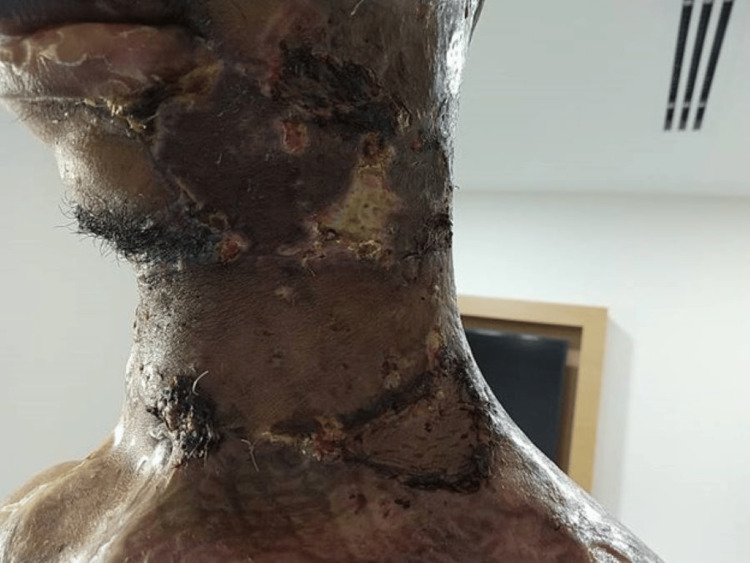
Image of the left side of the face and neck evident of disfigurement.

Following an approximately six-month hospital stay, the patient achieved stable wound healing and full clinical recovery. He was discharged in a stable yet disfigured condition with arrangements for ongoing outpatient rehabilitative and reconstructive care (Figures 12-14).

Objective severity scoring and outcome prediction

Objective burn severity scores were calculated to quantify injury severity and predicted mortality risk. In Case 1, the Abbreviated Burn Severity Index (ABSI) was 9, corresponding to an estimated mortality risk of approximately 30-50%. The Revised Baux score was calculated as 66 (age + TBSA), suggesting an estimated mortality risk of approximately 66% in conventional prediction models. In Case 2, the ABSI score was 7, corresponding to an estimated mortality risk of 10-20%, while the Revised Baux score was 71, corresponding to an estimated mortality risk of approximately 71%. Despite these high predicted mortality risks, both patients survived following aggressive multidisciplinary critical care, surgical intervention, and prolonged rehabilitation (Table 3).

**Table 3:** Abbreviated Burn Severity Index (ABSI) and revised Baux scores for both cases

Parameter	Case 1	Case 2
Age (years)	24	33
Sex	Female	Male
TBSA (%)	42%	38%
Full-thickness burn	Yes	Yes
Inhalation injury	No	No
ABSI Components		
Age score	2	2
Sex score	1	0
TBSA score	5	4
Full-thickness score	1	1
Inhalation score	0	0
Total ABSI score	9	7
Estimated mortality (ABSI)	~30-50%	~10-20%
Revised Baux score (age + TBSA ±17)	66	71
Estimated mortality (revised Baux)	~66%	~71%

## Discussion

Chemical burns resulting from intentional assault represent a particularly severe and complex subset of burn injuries, often characterized by extensive total body surface area involvement, deep tissue destruction, and early systemic complications. The two cases presented illustrate the profound local and systemic consequences of caustic exposure as well as the prolonged multidisciplinary care required for survival and functional recovery. Importantly, they highlight the need for early anticipation of systemic complications even in patients who initially appear hemodynamically stable.

Assault-related chemical burns consistently demonstrate a predilection for the face, neck, and upper body, reflecting the deliberate targeting of visible and functionally critical areas. In a 20-year retrospective study of chemical assault burns at a specialized burn center, Lin et al. reported that face and neck involvement was present in the majority of cases and was independently associated with airway complications and prolonged hospitalization [5]. A more recent global systematic review of acid and alkali assault burns similarly identified the face and neck as the most commonly affected anatomical regions, with extensive TBSA involvement and high rates of systemic complications reported across multiple centers [6]. The TBSA involvement in both of our cases (42% and 38%, respectively) substantially exceeds the global mean TBSA of 14.12% reported across chemical assault burn cases in the Warner-Levy et al. systematic review, whereby the face, head and eyes were the most commonly affected anatomical regions accounting for 39% of all body parts burned across reported cases, a pattern directly mirrored in both of our patients, in whom facial and neck involvement drove the most immediately life-threatening complication - airway compromise - from the outset [6]. This underscores that the cases presented in this series represent the severe end of the chemical assault burn spectrum. Lin et al. similarly noted a significantly prolonged hospitalization course in patients with high-TBSA chemical assault burns [5]. The six- and seven-month admissions in our cases are consistent with this reported trajectory and reflect the cumulative demands of serial debridement, staged reconstruction and systemic complication management that characterize this injury subset. This pattern was evident in both cases, in which burns involved the face, neck, and upper body bilaterally, placing both patients at immediate risk of airway compromise and systemic deterioration from the outset.

Airway compromise remains one of the most immediate and life-threatening concerns in severe chemical burns, particularly when the neck and oropharynx are involved. In both cases, early airway intervention was crucial, with intubation performed pre-emptively, due to impending obstruction. Notably, in Case 2, bilateral neck escharotomy was performed prior to intubation but failed to relieve progressive neck tightness and respiratory difficulty, underscoring that escharotomy alone is insufficient to secure the airway when circumferential cervical burns produce deep edema extending to the supraglottic structures. These cases reinforce the clinical recommendation that early elective intubation should be strongly considered in patients with circumferential neck burns or progressive edema rather than delaying intervention until overt respiratory distress develops, as the window for safe oral intubation narrows rapidly as edema progresses.

Beyond cutaneous injury, high-TBSA chemical burns are associated with profound systemic inflammatory responses. Burns exceeding 30% TBSA are known to precipitate burn shock, characterized by hypovolemia, capillary leak, myocardial depression, and increased systemic vascular resistance [4]. Despite early and appropriate fluid resuscitation guided by established burn formulas, both patients demonstrated systemic complications consistent with this pathophysiological cascade.

Rhabdomyolysis occurred in both patients and represented a clinically significant contributor to renal injury. Rhabdomyolysis is defined as the rapid destruction of skeletal muscle, with intracellular proteins and other toxic materials appearing in the bloodstream. These precipitate in the renal tubules and cause acute kidney injury that can result in death [7]. While rhabdomyolysis in thermal burns is primarily attributed to hypovolemia-driven ischemic muscle necrosis, chemical burns introduce an additional and distinct pathophysiological mechanism - the direct percutaneous penetration of caustic agents into deeper tissue compartments. Alkali agents, in particular, injure tissue through three concurrent processes: saponification of cell membrane lipids, which disrupts the skin’s permeability barrier and facilitates deeper penetration, alkaline hydrolysis of structural proteins causing liquefactive necrosis, and hygroscopic tissue dehydration compounded by exothermic reactions [8]. Unlike acid burns, which produce coagulative necrosis forming a self-limiting eschar, alkali-induced liquefactive necrosis does not restrict further tissue penetration, allowing the caustic agent to reach subcutaneous and deeper tissue compartments, including muscle, where direct cytotoxic injury triggers myocyte breakdown and the release of myoglobin, creatine kinase, and other intracellular contents into the systemic circulation. This mechanism of direct muscle injury operates independently of hemodynamic compromise, which may explain why both patients exhibited pigmenturia and early CK elevation despite being hemodynamically stable on presentation. In Case 1, CK was elevated at 196 U/L on admission, and urine output was maintained above 60 mL/hour throughout resuscitation, consistent with adequate renal perfusion; AKI was identified early but remained non-progressive. In Case 2, however, CK rose from 292 U/L on admission to 1,912 U/L by Day 7, reflecting ongoing muscle injury in the setting of extensive burns and systemic inflammatory amplification, ultimately resulting in progressive renal failure requiring CRRT. This trajectory is consistent with published data demonstrating that burned patients with rhabdomyolysis face substantially higher rates of AKI and mortality compared to non-burned patients with rhabdomyolysis [9]. The divergence in renal outcomes between the two cases, despite comparable initial CK values and similar resuscitation strategies, underscores that rhabdomyolysis severity in chemical burns may not be fully apparent on presentation and can evolve insidiously over the first week. This has direct clinical implications whereby serial CK monitoring beyond the initial resuscitative period and early nephrology involvement triggered by rising or persistently elevated values is essential in chemical burns exceeding 30% TBSA, even when early renal function appears preserved.

Acute kidney injury in extensive chemical burns represents the convergence of multiple simultaneous insults, and the renal trajectories observed in both cases illustrate this multifactorial pathophysiology clearly. The first and most direct mechanism is rhabdomyolysis-induced tubular injury, in which myoglobin released from injured muscle is freely filtered at the glomerulus and precipitates within the renal tubules in the setting of acidic urine, causing direct tubular toxicity, intra-luminal obstruction, and vasoconstriction of the afferent arteriole through myoglobin-mediated nitric oxide scavenging [7]. Superimposed on this is the hemodynamic insult of burn shock, which reduces effective circulating volume, decreases cardiac output, and increases renal vascular resistance, collectively reducing glomerular filtration rate even in patients who appear hemodynamically stable by conventional monitoring [4]. A third and clinically under-appreciated contributor is nephrotoxic medication exposure during the critical illness phase. Both patients received piperacillin-tazobactam as empiric broad-spectrum antibiotic therapy, an agent increasingly associated with acute tubular injury, particularly in the context of pre-existing renal impairment or concurrent hemodynamic instability. The cumulative nephrotoxic burden in critically ill burn patients, encompassing prolonged antibiotic courses and in some centers iodinated contrast from diagnostic imaging, compounds tubular injury already initiated by myoglobin and ischemia, and should be actively minimized through antibiotic stewardship and avoidance of nephrotoxic agents where alternatives exist. In Case 2, the combination of a rising CK trajectory, severe metabolic acidosis with pH 7.02 on admission, early creatinine elevation of 1.25 mg/dL, and ongoing systemic inflammatory burden created conditions in which progressive renal dysfunction was not preventable with standard resuscitation alone, and CRRT was initiated. The choice of CRRT over intermittent hemodialysis reflects established practice in hemodynamically unstable critically ill patients; by delivering gradual solute and fluid removal over 24 hours, CRRT avoids the rapid osmotic and intravascular volume shifts associated with intermittent sessions that are poorly tolerated in patients with vasopressor dependence or ongoing hemodynamic instability [10]. In the context of major burns, where fluid overload from aggressive resuscitation compounds pulmonary and wound edema, CRRT additionally allows for precise fluid balance management without the hemodynamic penalty of intermittent sessions. CRRT was subsequently discontinued prior to discharge, indicating the return of sufficient renal function - a clinically meaningful outcome given the severity of the admission profile. Based on the clinical trajectories in both cases, a structured renal surveillance protocol is warranted in all chemical burn patients with TBSA exceeding 30%. CK should be measured on admission and repeated every 12 to 24 hours for a minimum of seven days irrespective of initial values, urine output should be targeted at 0.5 to 1 mL/kg/hour during resuscitation, increased to 1 to 2 mL/kg/hour in the presence of confirmed pigmenturia, and nephrology consultation should be triggered proactively when CK exceeds 5,000 U/L, creatinine rises by more than 0.3 mg/dL within 48 hours, or urine output fails to meet targets despite adequate fluid administration [7, 10].

Coagulopathy emerged as a major systemic complication in both patients and was particularly severe and clinically consequential in Case 2. Burn-associated coagulopathy in major burns is a multifactorial process driven by several simultaneous and reinforcing pathways. The massive systemic inflammatory response triggered by extensive tissue destruction activates the extrinsic coagulation cascade through tissue factor release and cytokine-mediated endothelial injury, leading to widespread thrombin generation and progressive consumption of clotting factors [11]. Simultaneously, activation of fibrinolytic pathways accelerates the breakdown of fibrin clots, depleting fibrinogen and perpetuating a hemorrhagic tendency. Superimposed on this consumptive process, aggressive fluid resuscitation, while essential for hemodynamic stabilization and renal protection, contributes to dilutional coagulopathy by reducing circulating concentrations of clotting factors and platelets. Hypothermia, which is common in major burn patients due to loss of the skin barrier and prolonged operative exposure, further impairs enzymatic coagulation reactions and platelet function. The combined result of these processes in burns exceeding 30% TBSA is a coagulopathic state that closely resembles DIC, in which systemic coagulation activation occurs alongside depletion of endogenous anticoagulant mechanisms, ultimately producing both a bleeding tendency and the risk of microvascular thrombosis [11].

Applying the International Society of Thrombosis and Hemostasis (ISTH) DIC scoring system, the internationally validated diagnostic framework based on platelet count, PT prolongation, fibrin-related markers, and fibrinogen level, with a score of ≥5 defining overt DIC, both patients fulfilled criteria for overt DIC on admission [12]. Case 1 achieved an ISTH score of 6, derived from a markedly prolonged PT of 25.6 seconds (>6 seconds above normal, two points), strongly elevated D-dimer exceeding 20 µg/mL (three points), and a critically low fibrinogen of 87 mg/dL, below the 1 g/L threshold (one point). Case 2 achieved an ISTH score of 5, with a PT of 28.2 seconds (two points) and D-dimer exceeding 20 µg/mL (three points), with fibrinogen of 132 mg/dL remaining above the scoring threshold at presentation, though subsequent clinical deterioration with progressive thrombocytopenia - platelet counts declining from 194 × 10³/µL to approximately 131 × 10³/µL - reflected ongoing consumptive coagulopathy (Table 4).

**Table 4:** ISTH overt DIC scoring applied to both cases Scored per Taylor et al. (Thromb Haemost, 2001). A score of ≥5 is compatible with overt DIC. ISTH: International Society of Thrombosis and Hemostasis, DIC: disseminated intravascular coagulation

Parameter	Scoring threshold	Case 1 value	Points	Case 2 value	Points
Platelet count	>100 = 0 50–100 = 1 <50 = 2	Normal on admission	0	194 × 10³/µL (normal)	0
D-dimer / fibrin marker	No increase = 0, Moderate = 2, Strong = 3	>20 µg/mL FEU (markedly elevated)	3	>20 µg/mL FEU (markedly elevated)	3
PT prolongation	<3s = 0 3–6s = 1 >6s = 2	25.6 s (>6 s above normal)	2	28.2 s (>6 s above normal)	2
Fibrinogen	>1 g/L = 0 <1 g/L = 1	87 mg/dL (0.87 g/L — below threshold)	1	132 mg/dL (1.32 g/L — above threshold)	0
Total ISTH score		Score: 6	6	Score: 5	5
Interpretation (≥5 = overt DIC)		Overt DIC		Overt DIC	

The severity of coagulopathy in Case 2 had direct implications for surgical planning. Definitive wound coverage and grafting could not be safely undertaken until coagulation parameters had been stabilized through aggressive component therapy, contributing to the interval between injury and the reconstructive phase. Over approximately three months of critical illness, the patient received 63 units of packed red blood cells, 24 units of fresh frozen plasma, and one unit of platelets, a transfusion burden that reflects the sustained hemostatic instability characteristic of extensive chemical burns. This experience reinforces the recommendation that coagulation screening, including PT, INR, aPTT, fibrinogen, and D-dimer, should be obtained on admission and repeated at 24-48 hour intervals during the first week in any chemical burn exceeding 30% TBSA, with early hematology input and a proactive component therapy strategy guided by serial laboratory trends rather than reactive transfusion alone [11].

The development of multi-organ dysfunction in these patients was likely multifactorial and reflects the unique pathophysiological profile of high-TBSA chemical burns. Extensive TBSA involvement exceeding 30-40% is associated with systemic inflammatory response activation, capillary leak, and cytokine-mediated endothelial dysfunction [4]. In chemical burns specifically, ongoing tissue penetration and protein denaturation - particularly with alkali agents, which cause liquefactive rather than self-limiting coagulative necrosis - may result in deeper injury than initially apparent, amplifying inflammatory and metabolic stress beyond what the surface area of injury alone would predict [3, 8]. This helps explain why both patients, despite presenting hemodynamically stable with apparently contained injuries, rapidly developed systemic complications, including rhabdomyolysis, consumptive coagulopathy, and metabolic acidosis within hours of presentation. The systemic inflammatory cascade triggered by extensive chemical injury likely contributed to each of the end-organ insults observed: rhabdomyolysis through direct cytotoxic muscle injury and ischemia, AKI through the convergence of myoglobin-induced tubular toxicity, reduced renal perfusion from burn shock and nephrotoxic medication exposure, and coagulopathy through tissue factor-driven coagulation activation, fibrinolysis, and dilutional hemostatic impairment from resuscitation. Although no delay in presentation was documented in either case, the severity and depth of chemical exposure were sufficient to trigger widespread physiological derangement despite prompt resuscitative measures. The presence of deep dermal and full-thickness burns in critical anatomical areas such as the face and neck, combined with early biochemical derangements including severe metabolic acidosis, markedly elevated D-dimer, and rising CK, may serve as early warning indicators of impending multi-organ dysfunction and should prompt intensification of monitoring and proactive specialist involvement even when hemodynamic parameters remain within normal limits.

Surgical management of chemical burns poses unique challenges due to ongoing tissue injury and delayed wound demarcation. Both patients required serial debridement over several weeks to months, reflecting the progressive nature of chemical tissue necrosis and the unpredictable evolution of apparent tissue viability in the days following caustic exposure. Unlike thermal burns, in which burn depth and wound margins tend to declare themselves within 48-72 hours, chemical burns - particularly from alkali agents - may continue to penetrate tissue for a prolonged period, meaning that apparently viable tissue at initial assessment may subsequently declare itself non-viable [8]. In both cases, the decision to proceed to definitive grafting was guided by the achievement of a clean, well-vascularized wound bed on serial assessment, combined with hematological and hemodynamic stability sufficient to tolerate operative intervention safely. Definitive wound coverage was achieved through staged split-thickness and full-thickness skin grafting once wound beds were optimized. Contracture release procedures, including Z-plasty and eyelid reconstruction, were required to restore function and prevent long-term disability, consistent with well-documented reconstructive challenges in chemical burn survivors when joints, the axilla, or periocular regions are involved. The prolonged reconstructive timeline in both cases reflects the need for individualized surgical planning and long-term multidisciplinary follow-up.

Infection represents a major risk in patients with extensive burns due to loss of the skin barrier, systemic immunosuppression and prolonged hospitalization. Both cases were complicated by wound infections with multidrug-resistant organisms, including Pseudomonas aeruginosa and AmpC-producing Klebsiella pneumoniae in Case 1, though neither patient progressed to septic shock. Antimicrobial therapy was tailored according to wound culture and susceptibility results in both cases, underscoring the importance of systematic wound surveillance, early microbiological sampling, and coordinated infectious disease input in this population.

A multidisciplinary approach was central to the management and recovery of both patients. In addition to burn and plastic surgeons, care involved intensivists, ophthalmologists, nephrologists, nutritionists, physiotherapists, and psychologists. Severe chemical assault burns carry a significant psycho-social burden, and early psychological intervention is essential to address long-term mental health outcomes, including post-traumatic stress disorder, depression, and anxiety, which are disproportionately prevalent in assault-related burn survivors compared to accidental burn populations.

Despite similarities in injury mechanism and anatomical distribution, the two cases demonstrated marked variability in systemic severity and recovery trajectories. While both patients required prolonged ICU care and multiple reconstructive procedures, Case 2 developed more severe metabolic acidosis, progressive rhabdomyolysis, AKI requiring CRRT, and a substantially greater transfusion requirement, despite a lower TBSA and younger age than would predict such severity. This heterogeneity highlights that TBSA alone is insufficient to predict systemic complication burden in chemical burns, and that the depth of tissue penetration, the nature of the caustic agent, and the early biochemical response - particularly CK trajectory, acid-base status, and coagulation profile within the first 24 to 48 hours - may be more informative markers of impending multi-organ dysfunction.

Prognostic significance of burn severity indices

Objective burn severity scoring systems are widely used to estimate mortality risk and guide clinical decision-making in major burn injuries. In the present cases, both patients demonstrated high predicted mortality based on established burn severity indices, including the ABSI and revised Baux score. However, survival outcomes were significantly better than predicted mortality models, highlighting the impact of modern advances in critical care, early airway management, aggressive resuscitation protocols, and multidisciplinary surgical reconstruction.

Case 1 demonstrated a higher ABSI score compared to Case 2, primarily due to female sex and extensive full-thickness burn involvement. However, Case 2 demonstrated a higher Revised Baux score due to older age and similar TBSA involvement. The discrepancy between scoring systems reflects differences in prognostic weighting between demographic factors and physiological injury burden.

The survival of both patients despite high mortality risk scores supports evidence that traditional prognostic models may overestimate mortality in settings where advanced ICU support, early surgical intervention, and aggressive transfusion strategies are available. This finding is consistent with contemporary burn literature demonstrating improved survival outcomes in high-TBSA burn patients managed in specialized burn centers.

Validated scales to quantify long-term impact

Long-term outcomes in severe chemical burn survivors are commonly quantified using validated instruments such as the Burn Specific Health Scale-Brief (BSHS-B), the Disabilities of the Arm, Shoulder and Hand (DASH) score, and the Patient and Observer Scar Assessment Scale (POSAS). Although formal scoring was not performed in the present cases due to follow-up constraints, incorporation of standardized outcome measures in future studies would allow objective assessment of functional recovery, scar quality, and psychosocial impact.

## Conclusions

These cases demonstrate that severe assault-related chemical burns involving greater than 30% TBSA carry a high risk of early multi-organ dysfunction, including rhabdomyolysis, overt disseminated intravascular coagulation, and progressive acute kidney injury, even in patients who present in a hemodynamically stable condition. The divergent renal trajectories between the two cases - preserved renal function in Case 1 despite early pigmenturia, versus progressive AKI requiring CRRT in Case 2 despite equivalent resuscitation targets - underscore that initial CK values and hemodynamic stability are insufficient predictors of renal outcome in extensive chemical burns. Serial CK monitoring, early coagulation screening, and proactive nephrology and hematology involvement should be initiated from the time of admission rather than deferred until overt organ dysfunction declares itself.

From an airway management perspective, these cases reinforce that circumferential cervical burns with facial involvement represent a time-critical situation in which the window for safe oral intubation narrows rapidly. The failure of neck escharotomy alone to secure the airway in Case 2 highlights that deep supraglottic edema may develop independently of eschar tension, and early elective intubation should not be delayed in anticipation of escharotomy effect.

Survival in both cases, despite predicted mortality risks of 30-71% based on ABSI and revised Baux scoring, reflects the impact of aggressive multidisciplinary critical care, early surgical intervention, and sustained reconstructive commitment over months of hospitalisation. Future studies incorporating standardized long-term outcome measures such as the BSHS-B and POSAS would allow more objective characterization of functional and quality-of-life recovery in this patient population.

Documenting such cases not only contributes to the evolving understanding of chemical burn pathophysiology and outcomes but also highlights the urgent need for standardized management frameworks and broader preventive strategies to address this severe manifestation of interpersonal violence.
